# Hospital-recorded congenital syphilis rates by maternal origin in a referral hospital in Quito, Ecuador, 2009–June 2024: a retrospective observational study

**DOI:** 10.3389/frph.2026.1985888

**Published:** 2026-10-30

**Authors:** Paola Toapanta-Pinta, Cristhian Vasco-Toapanta, Verónica Oliva-Velasco, Mónica Díaz-Torres, Santiago Vasco-Morales

**Affiliations:** 1 Facultad de Ciencias Médicas, Universidad Central del Ecuador, Quito, Ecuador; 2 Grupo de Investigación en Salud Infantil y Perinatal (ISIP), Dirección de Investigación, Universidad Central del Ecuador, Quito, Ecuador; 3 Posgrado de Pediatría, Universidad Internacional del Ecuador, Quito, Ecuador; 4 Servicio de Pediatría, Hospital Metropolitano, Quito, Ecuador; 5 Servicio de Neonatología, Hospital Gineco-Obstétrico Isidro Ayora, Quito, Ecuador; 6 Escuela de Medicina, Universidad San Francisco de Quito, Quito, Ecuador; 7 Servicio de Neonatología, Hospital Gineco-Obstétrico Pediátrico de Nueva Aurora, Quito, Ecuador

**Keywords:** Bayes theorem, Ecuador, emigrants and immigrants, human migration, prenatal care, congenital syphilis

## Abstract

**Background:**

Congenital syphilis is preventable, yet gaps in prevention persist during pregnancy. In Ecuador, intensified migration highlights the need to examine differences by maternal country of origin. We evaluated the association between maternal origin and rates of hospital-recorded congenital syphilis, temporal patterns, and, exploratorily, prenatal visits among recorded cases.

**Methods:**

Retrospective observational study used records from a tertiary referral hospital in Quito, Ecuador, from January 2009 through June 2024. Live-birth denominators by maternal origin came from the census, and cases were identified in the Perinatal Information System among hospitalized newborns. The primary analysis comprised 32 strata defined by calendar period and maternal origin and used Bayesian Poisson regression with a flexible temporal function and live births as an offset. The secondary analysis was restricted to recorded cases.

**Results:**

Among 111,742 live births, 255 congenital syphilis cases were recorded: 219 (85.9%) among mothers of Ecuadorian origin and 36 (14.1%) among mothers of foreign origin. Births to mothers of foreign origin had a higher recorded-case rate (RR = 2.47; 95% CrI: 1.69–3.51). Rates followed a nonlinear pattern, peaking in this group in 2018. Among 250 cases in the exploratory analysis, the expected number of prenatal visits was lower with foreign maternal origin (MR = 0.797; 95% CrI: 0.654–0.963).

**Conclusions:**

Births to mothers of foreign origin showed higher rates of hospital-recorded congenital syphilis and, among recorded cases, fewer expected prenatal visits. The temporal coincidence with migration trends is descriptive and does not establish that migration or irregular migration status explains these differences.

## Introduction

Congenital syphilis is preventable through timely detection of maternal infection and adequate treatment during pregnancy. However, missed opportunities persist in relation to absent or delayed screening, delayed entry into prenatal care, and absent or inadequate maternal treatment. Moreover, contact with prenatal services alone does not guarantee timely implementation of interventions necessary to prevent vertical transmission (1, 2).

Maternal origin is a relevant characteristic for examining differences in the occurrence of congenital syphilis. Recent epidemiologic surveillance data have shown differences by maternal country of birth and an increase in cases among mothers born outside the country in certain contexts (3). Nevertheless, country of origin, country of birth, nationality, and migration status are distinct concepts and should not be interpreted interchangeably or as independent indicators of social vulnerability or access to health care.

In Ecuador, prevention gaps related to syphilis during pregnancy have been documented, including incomplete screening coverage and delayed prenatal screening (4). Moreover, national hospital discharge records have shown differences in obstetric complications among Venezuelan refugee and migrant women compared to Ecuadorian women, including a grouped category of sexually transmitted infections, although not specifically syphilis (5).

During the study period, Ecuador experienced a marked increase in Venezuelan migration amid the socioeconomic and humanitarian crisis in Venezuela, evolving from primarily a transit country to an important destination country; by September 2019, nearly 400,000 Venezuelans had settled in Ecuador. Irregular migration status was also common, referring to the absence of regular authorization to remain in the country rather than necessarily to entry through unofficial border crossings (6, 7). This scenario provides a relevant epidemiologic context to examine the evolution of congenital syphilis according to maternal origin without assuming that foreign origin equates to irregular migration status.

Available data from Ecuador have not characterized the rate of hospital-recorded congenital syphilis cases according to maternal origin using live-birth denominators specific to each group or described their evolution over a prolonged period (4, 5). Therefore, the primary objective was to estimate the association between maternal origin and the rate of hospital-recorded congenital syphilis cases per 1,000 institutional live births from January 2009 through June 2024, as well as to describe its temporal evolution. In an exploratory analysis, we also evaluated differences in the number of prenatal visits according to maternal origin among recorded cases.

## Methods

### Study design and setting

A retrospective observational study was conducted using institutional records from the Hospital Gineco-Obstétrico Isidro Ayora, a tertiary-level public referral hospital in Quito, Ecuador, from January 2009 through June 2024.

The study included two components. The primary analysis used aggregated information by calendar period and maternal origin to evaluate the rate of hospital-recorded congenital syphilis cases. Thirty-two strata were defined corresponding to 16 calendar periods and two maternal origin categories; the 2024 strata comprised only January–June. The secondary analysis used individual-level information and was restricted to recorded congenital syphilis cases.

### Population, data sources, and selection

The denominators for the primary analysis comprised all live births occurring at the institution and were obtained from the institutional census of births by calendar period and maternal origin. Congenital syphilis cases were identified in the Perinatal Information System (SIP), which contained individual information on hospitalized newborns and their mothers. Therefore, while the denominator included all institutional live births, case identification was restricted to hospitalized newborns recorded in the SIP.

The primary analysis included all live births recorded in the institutional census with available information on maternal origin. All 255 recorded congenital syphilis cases had available information on maternal origin and corresponding institutional live-birth denominators; therefore, no eligible cases were excluded from the primary analysis. The secondary analysis of prenatal visits required cases with available information on maternal origin, number of prenatal visits, and gestational age; five cases were excluded because of missing gestational age data.

### Study size

All eligible records from January 2009 through June 2024 were included; no prospective sample size calculation was performed.

The selection of recorded congenital syphilis cases for the primary analysis is summarized in Supplementary Figure S1.

For each period, the number of live births to mothers of Ecuadorian origin was calculated as the difference between the institutional total and recorded births to mothers of foreign origin. Historical data sources did not permit separate identification of records with missing or undetermined maternal origin (Supplementary Table S1).

### Case definition and maternal origin

Congenital syphilis cases were identified among hospitalized newborns whose final discharge diagnosis recorded in the SIP specifically documented “congenital syphilis.” This diagnosis reflected the clinical assessment completed during hospitalization rather than neonatal seroreactivity alone. However, the study did not independently verify the diagnostic criteria applied to each case or retrospectively reclassify cases using contemporary clinical or serologic criteria, maternal test results, or treatment information. Consequently, temporal variation in diagnostic or recording practices and some degree of diagnostic misclassification cannot be excluded.

Maternal origin was derived from the country-of-origin field in the institutional records. Ecuador was classified as Ecuadorian origin and all other countries as foreign origin. Country-specific information was available for the aggregated institutional live-birth denominators; however, the anonymized individual-level analytic dataset retained maternal origin only as Ecuadorian or foreign. Consequently, country-specific congenital syphilis case counts and rates could not be estimated. Maternal origin was not interpreted as equivalent to nationality, migration status, duration of residence, or social vulnerability.

### Efforts to address bias

Institutional census data provided complete live-birth denominators for each maternal-origin category. However, case ascertainment was restricted to hospitalized newborns recorded in the SIP. Consequently, differential hospitalization, diagnosis, or recording according to maternal origin could result in differential case ascertainment and bias the estimated rate ratio; the direction and magnitude of such bias could not be determined from the available data. Temporal variation in diagnostic coding or recording practices was addressed through sensitivity analyses excluding selected periods (2021 and 2024 strata) and examining robustness to different prior distributions. Unmeasured confounding by social determinants, healthcare access barriers, and other maternal factors was conceptualized using a directed acyclic graph (Figure 1) as an interpretive framework, rather than defined as a covariate adjustment set, because individual-level covariates were unavailable for all live births contributing to the denominators.

**Figure 1:**
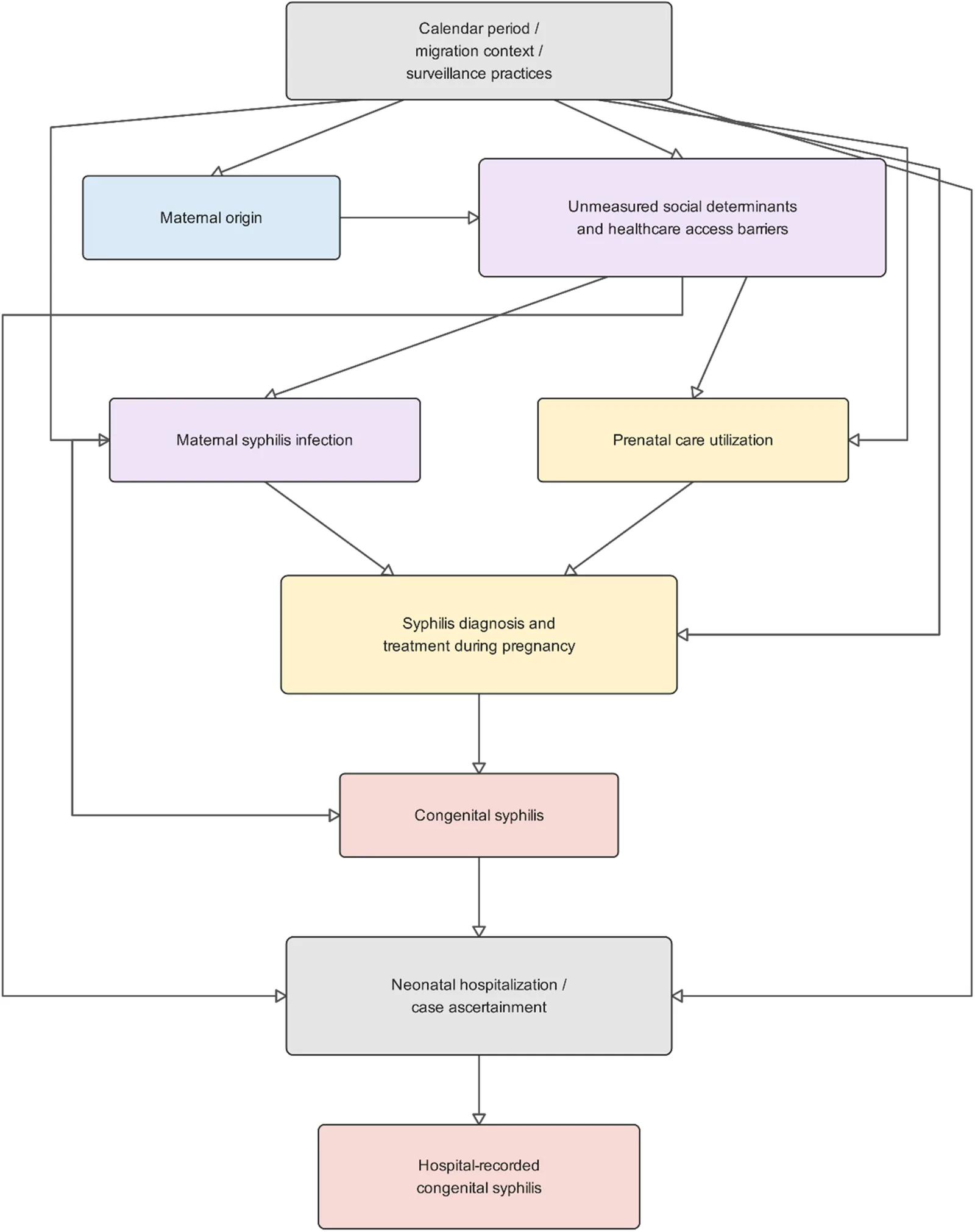
Conceptual directed acyclic graph for interpretation of the maternal-origin contrast in hospital-recorded congenital syphilis. The DAG summarizes hypothesized relationships among maternal origin, social determinants and healthcare access barriers, maternal syphilis, prenatal care, and hospital case ascertainment. It is intended as an interpretive framework rather than to define a covariate adjustment set because individual-level determinants were unavailable for the population contributing the live-birth denominators. Prenatal care information was available only among recorded congenital syphilis cases; therefore, its potential mediating role could not be assessed at the population level.

**Figure 2:**
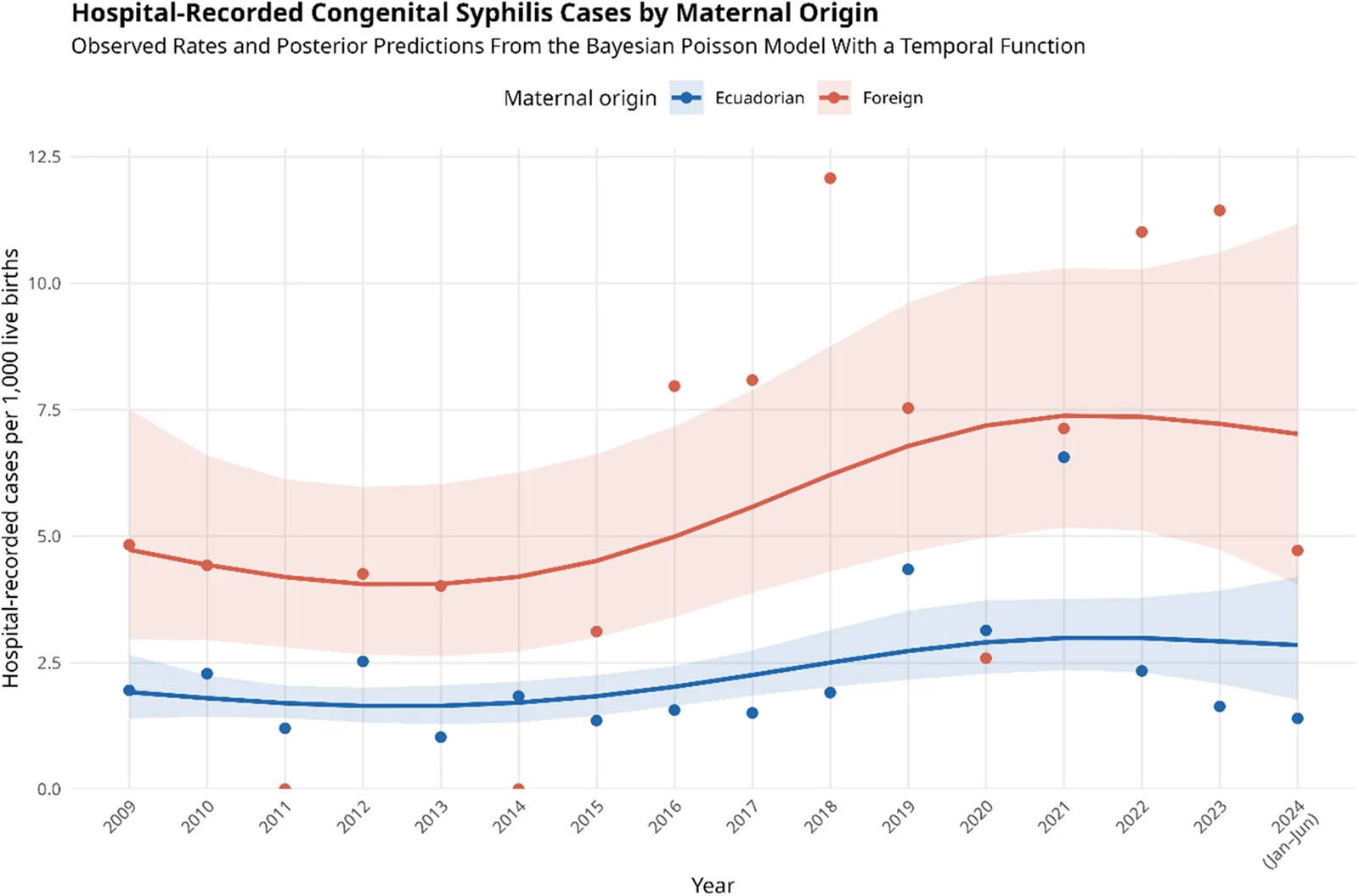
Observed rates and posterior predictions of hospital-recorded congenital syphilis cases by maternal origin, 2009–June 2024. Points represent observed rates per 1,000 institutional live births. Lines represent posterior expected rates estimated using the Bayesian Poisson regression model with a thin-plate spline function of calendar time and the logarithm of live births as an offset; shaded bands represent 95% credible intervals. The maternal-origin contrast was modeled as constant over time. Data for 2024 include January through June only.

### Variables and descriptive analysis

Among cases, we analyzed maternal age, number of prenatal visits, gestational age, and birth weight, as well as stable partnership, planned pregnancy, educational attainment, prematurity, small for gestational age, delivery type, maternal hypertensive disorder, congenital anomalies, and neonatal death.

Prenatal care density was calculated as prenatal visits divided by weeks of gestation and was used only for descriptive purposes. Quantitative variables were summarized using median and interquartile range (IQR), and categorical variables using frequencies and percentages. Percentages were calculated on records with available information; missing data were not imputed.

For each stratum, the observed rate was calculated as the number of hospital-recorded cases divided by the corresponding number of institutional live births, expressed per 1,000 live births.

### Primary statistical analysis

We explored temporal structure, dispersion of counts, and presence of influential observations. Poisson models with different temporal specifications were compared with a negative binomial model; the Poisson family was selected for parsimony and absence of relevant predictive improvement with the more flexible alternative. Detailed exploratory analyses are presented in the supplementary material.

The primary analysis used Bayesian Poisson regression for the number of cases in each stratum. The model included maternal origin as the primary exposure, a temporal function via a thin-plate spline, and the logarithm of live births as an offset. A constant contrast between maternal origin categories was assumed, without origin-time interaction or prespecified change points.

A Normal(−6, 1.5) prior distribution was used for the intercept and a Normal(0, 1) prior for the maternal origin coefficient. The association was expressed as a rate ratio (RR), 95% credible interval (95% CrI), and posterior probability that RR > 1.

Individual maternal covariates were not incorporated because they were not equivalently available for the entire population used as the denominator. Therefore, estimation was adjusted for temporal structure but not for potential individual maternal confounders. A conceptual directed acyclic graph (DAG) was used to summarize the hypothesized relationships relevant to interpretation of the maternal-origin contrast, including unmeasured social determinants and healthcare access barriers, maternal syphilis, prenatal care, and case ascertainment (Figure 1). The DAG was used as an interpretive framework rather than to define a covariate adjustment set.

Posterior sampling used Markov chain Monte Carlo (MCMC) with four chains of 4,000 iterations, including 2,000 warmup iterations, with seed 20260810, adapt_delta = 0.999, and max_treedepth = 15.

### Diagnostics and sensitivity analysis

Convergence and sampling efficiency were evaluated using R-hat, effective sample sizes, divergences, max_treedepth, and E-BFMI. Predictive adequacy was examined via posterior predictive checks and leave-one-out cross-validation (LOO) with Pareto-k. Complete diagnostic results are presented in the supplementary material.

Robustness was evaluated by refitting the model after excluding 2021 strata due to their exploratory influence and 2024 strata due to partial observation. Sensitivity to different prior distributions for the maternal origin coefficient was also examined.

### Exploratory secondary analysis of prenatal care

The secondary analysis was restricted to cases with available information on maternal origin, number of prenatal visits, and gestational age. The number of visits was analyzed as count data, and Bayesian Poisson, negative binomial, and zero-inflated variants were compared via posterior predictive adequacy and LOO.

A zero-inflated Poisson model adjusted for gestational age at delivery was selected; gestational age was included to represent the cumulative opportunity to receive prenatal visits and not as a confounder adjustment. The structural zero component used a common intercept. The association with maternal origin was expressed as mean ratio (MR), 95% CrI, and corresponding posterior probability.

A Normal(log(6), 1) prior distribution was used for the intercept of the count component, Normal(0, 1.5) for coefficients, and Beta(1, 1) for zero inflation. Sampling used four chains of 3,000 iterations, including 1,500 warmup iterations, with adapt_delta = 0.97 and max_treedepth = 13.

The proportion with ≥8 visits and prenatal care density were used exclusively for descriptive purposes. Because this analysis was conditioned on the presence of congenital syphilis, its results describe associations among cases and were not interpreted as population estimates or as evidence of mediation by prenatal care. This case-restricted conditioning and its implications for interpretation are represented conceptually in Figure 1.

Analyses were conducted in R 4.5.0; Bayesian models were fitted with brms 2.23.0 and rstan 2.32.7, and posterior 1.7.0 and loo 2.8.0 were used for post-processing and cross-validation.

### Ethical considerations

The study was approved by the Research Ethics Committee for Human Subjects at the Hospital General Docente de Calderón (CEISH-HGDC-2024-007). Due to its retrospective design based on institutional records, a waiver of informed consent was granted, and data were managed according to confidentiality requirements established in the ethical approval.

## Results

### Study population and case characteristics

From January 2009 through June 2024, the institutional census recorded 111,742 live births, of which 106,042 occurred to mothers of Ecuadorian origin and 5,700 to mothers of foreign origin. Among the 5,700 live births to mothers of foreign origin, 3,036 (53.3%) were recorded as Venezuelan origin, 1,607 (28.2%) as Colombian origin, and 1,057 (18.5%) as other recorded origins (Supplementary Table S2). Country-specific information was available only for the aggregated live-birth denominators. In the SIP, 255 recorded congenital syphilis cases were identified; 219 cases (85.9%) occurred among births to mothers of Ecuadorian origin and 36 cases (14.1%) among births to mothers of foreign origin. Maternal origin was available in all 255 cases (100%).

Cases and live births were distributed across 32 strata defined by the combination of calendar period and maternal origin; the two strata corresponding to 2024 included only January–June. The denominators corresponding to each stratum are presented in Supplementary Table S1.

Maternal, prenatal, obstetric, and neonatal characteristics of cases are presented in Table 1.

**Table 1:** Maternal, obstetric, and neonatal characteristics of congenital syphilis cases by maternal origin.

Characteristic	Total	Ecuadorian origin	Foreign origin
Cases, n	255	219	36
Maternal age, median [IQR]	26	25	26.5
	[21.00–32.00]	[21.00–32.00]	[21.00–30.25]
Stable partnership, *n* (%)	179 (70.20%)	154 (70.32%)	25 (69.44%)
Planned pregnancy, *n* (%)	82 (32.16%)	68 (31.05%)	14 (38.89%)
Educational attainment, *n* (%)			
None	8 (3.14%)	8 (3.65%)	0 (0.00%)
Primary	74 (29.02%)	62 (28.31%)	12 (33.33%)
Secondary	158 (61.96%)	136 (62.10%)	22 (61.11%)
Higher	15 (5.88%)	13 (5.94%)	2 (5.56%)
Prenatal visits, median [IQR]	6	6	5
	[4.00–8.00]	[4.00–8.00]	[1.00–6.00]
Prenatal care density, median [IQR]	0.15	0.16	0.13
	[0.10–0.21]	[0.11–0.21]	[0.03–0.16]
Gestational age (weeks), median [IQR]	39	39	38
	[37.00–39.00]	[37.00–39.00]	[37.00–39.00]
Preterm, *n* (%)	32 (12.60%)	28 (12.84%)	4 (11.11%)
Birth weight (g), median [IQR]	2,925	2,940	2,905
	[2,616.00–3,232.50]	[2,620.00–3,280.00]	[2,554.25–3,155.00]
Small for gestational age, *n* (%)	54 (21.18%)	47 (21.46%)	7 (19.44%)
Cesarean delivery, *n* (%)	105 (41.18%)	93 (42.47%)	12 (33.33%)
Maternal hypertensive disorder, *n* (%)	28 (11.07%)	26 (11.98%)	2 (5.56%)
Congenital anomaly, *n* (%)	21 (8.24%)	20 (9.13%)	1 (2.78%)
Neonatal death, *n* (%)	2 (0.78%)	2 (0.91%)	0 (0.00%)

IQR, interquartile range. Prenatal care density was calculated as the number of prenatal visits divided by weeks of gestation and was used for descriptive purposes only. Percentages were calculated using cases with available data for each variable as the denominator; missing values were not imputed. Available observations were 251 for prenatal visits, 250 for prenatal care density, 254 for gestational age and preterm birth, and 253 for maternal hypertensive disorder; all other variables included 255 cases.

### Observed rates

Hospital-recorded congenital syphilis case rates varied between 1.03 and 6.56 cases per 1,000 live births among births to mothers of Ecuadorian origin and between 0 and 12.08 cases per 1,000 live births among births to mothers of foreign origin. Maximum values were observed in 2021 for Ecuadorian origin and in 2018 for foreign origin. Cases, denominators, and rates corresponding to each calendar period and maternal origin category are presented in Table 2; values for 2024 represent only the January–June period.

**Table 2:** Hospital-recorded congenital syphilis cases, institutional live births, and observed rates per 1,000 live births, by calendar period and maternal origin, 2009–June 2024.

Year	Ecuadorian origin			Foreign origin		
	Cases, n	Live births, n	Rate per 1,000	Cases, n	Live births, n	Rate per 1,000
2009	22	11,265	1.95	1	207	4.83
2010	26	11,392	2.28	1	226	4.42
2011	13	10,804	1.20	0	245	0.00
2012	24	9,507	2.52	1	235	4.26
2013	10	9,731	1.03	1	249	4.02
2014	16	8,710	1.84	0	213	0.00
2015	10	7,378	1.36	1	321	3.12
2016	9	5,757	1.56	2	251	7.97
2017	8	5,312	1.51	3	371	8.09
2018	9	4,717	1.91	5	414	12.08
2019	16	3,681	4.35	4	531	7.53
2020	14	4,463	3.14	2	773	2.59
2021	24	3,657	6.56	4	561	7.13
2022	9	3,851	2.34	5	454	11.01
2023	6	3,671	1.63	5	437	11.44
2024^a^	3	2,146	1.40	1	212	4.72

Rate was calculated as the number of hospital-recorded congenital syphilis cases divided by the number of institutional live births for the corresponding stratum and expressed per 1,000 live births. The evolution of observed rates and posterior model predictions is presented in Figure 2.

a Data for 2024 include only January through June.

Exploratory evaluation supported a nonlinear temporal structure; comparison of temporal specifications and dispersion statistics are presented in Supplementary Table S3. The negative binomial specification with the same temporal structure and offset showed no clear predictive improvement over the Poisson model (*Δ*ELPD = 2.64; SE = 5.30), so the Poisson family was retained for parsimony.

### Primary model and diagnostics

In the Bayesian Poisson regression model, births to mothers of foreign origin showed a higher rate of hospital-recorded congenital syphilis cases than births to mothers of Ecuadorian origin (RR = 2.47; 95% CrI: 1.69–3.51; P[RR > 1] > 0.9999). The estimate corresponds to the contrast between the two maternal origin categories during the study period, considering the common temporal structure specified in the model.

Maximum R-hat was 1.0021, and no divergences or max_treedepth exceedances were recorded (Supplementary Table S4). Posterior predictive checks reproduced overall dispersion, although the 2021 Ecuadorian origin stratum fell in the upper predictive tail (observed = 24; posterior median = 11; 95% posterior predictive interval: 5–18; Supplementary Table S5 and Supplementary Figure S2). All Pareto-k values were < 0.70; the maximum corresponded to the 2021–Ecuadorian origin stratum (k = 0.695; Supplementary Table S6).

### Sensitivity analysis

The primary association maintained the same direction in sensitivity analyses. When the two 2021 strata were excluded, the RR was 2.83 (95% CrI: 1.86–4.17; P[RR > 1] = 1.0000). When the two 2024 strata were excluded, the RR was 2.45 (95% CrI: 1.65–3.47; P[RR > 1] = 1.0000), compared to 2.47 (95% CrI: 1.69–3.51) in the primary model (Supplementary Table S7).

Estimates obtained with different prior distributions for the maternal origin coefficient ranged from RR = 2.27 to RR = 2.52, maintaining the same direction as the primary estimate (Supplementary Table S8).

### Exploratory secondary analysis of prenatal care

The number of prenatal visits was available in 251 of 255 cases (98.4%); the adjusted model included 250 cases with additional information on gestational age. Among cases with available prenatal visit information, the median was 6 visits (IQR: 4–8), with a range of 0–15, and 11 cases (4.4%) had no recorded prenatal visits.

Poisson and negative binomial models without zero inflation did not adequately reproduce the observed frequency of zeros in posterior predictive checks, so zero-inflated specifications were evaluated. The predictive difference between the zero-inflated Poisson and zero-inflated negative binomial models was small and imprecise (*Δ*ELPD = −1.34; SE = 3.61), so the zero-inflated Poisson model was selected for parsimony.

In the model adjusted for gestational age at delivery, the expected number of prenatal visits was lower among cases of foreign maternal origin than among those of Ecuadorian origin (MR = 0.797; 95% CrI: 0.654–0.963; P[MR < 1] = 0.992).

Descriptively, 66 of 215 cases of Ecuadorian origin (30.7%) and 7 of 36 cases of foreign origin (19.4%) recorded eight or more prenatal visits. The median prenatal care density was 0.158 visits per week (IQR: 0.106–0.205) among cases of Ecuadorian origin and 0.128 (IQR: 0.027–0.159) among those of foreign origin.

## Discussion

In this referral hospital, births to mothers of foreign origin showed a higher rate of hospital-recorded congenital syphilis cases than births to mothers of Ecuadorian origin. The association remained similar in direction and magnitude in sensitivity analyses excluding 2021 and the January–June 2024 period, as well as across different prior specifications. Rates exhibited a nonlinear temporal pattern, and among births to mothers of foreign origin, the highest rate was observed in 2018. This increase occurred during a period of intensification in migration flows to Ecuador, within a context of high levels of irregular migration (6, 7). This temporal coincidence provides epidemiologic context but does not allow the observed increase to be attributed to migration or migration status, neither of which was available at the individual level. In the exploratory analysis restricted to recorded cases, the expected number of prenatal visits was lower among those of foreign maternal origin after accounting for gestational age at delivery. This result describes an association within the set of cases and does not establish that prenatal care explains or mediates the observed difference in rates by maternal origin.

Similar findings have been reported in other settings. Studies in other countries have described differences in the frequency of congenital syphilis by maternal origin, while research among migrant populations has documented syphilis during pregnancy (3, 8, 9). However, these studies used different criteria to define origin or migration status, so their results are not directly comparable with ours. Consequently, the higher rate observed among births to mothers of foreign origin should not be attributed to maternal origin *per se*.

Country of origin may coexist with unmeasured social, administrative, and health service conditions. Fragmentation of services, administrative barriers, and difficulties in continuity of clinical information related to congenital syphilis prevention have been documented (10). Among Venezuelan migrant women in Colombia, syphilis was also associated with vulnerabilities such as food insecurity and lack of health service utilization; the association with irregular migration status observed in the unadjusted analysis was attenuated after adjustment and was not specific to pregnant women (8). Additionally, differences in obstetric complications by maternal origin have been described, including a grouped category of nonspecific sexually transmitted infections (5). These findings provide context for our results but do not allow such conditions to be attributed to the foreign-origin mothers in our study or to be considered explanatory mechanisms for the observed association. From a broader social and health-system perspective, congenital syphilis may also reflect cumulative barriers across the continuum of prevention, including timely access to care, continuity of care, screening, and treatment. In Quito, health practitioners have reported barriers to sexual and reproductive healthcare among Venezuelan migrant women, including socioeconomic vulnerability and difficulties related to migration documentation (11). National hospital-discharge data from Ecuador have also identified differences in obstetric complications and diagnosed sexually transmitted infections between Venezuelan refugee and migrant women and Ecuadorian women (5). These findings provide relevant contextual evidence of social and health-system inequalities; however, these conditions were not measured at the individual level in our study and therefore cannot be assumed to explain the association observed with maternal origin.

Missed opportunities in vertical transmission prevention may occur at various stages, including late entry into prenatal care, absent or delayed screening, and absent or inadequate maternal treatment (1, 2). Additionally, lower availability of rapid tests in primary care has been ecologically associated with higher incidence of congenital syphilis (12). These findings demonstrate that contact with prenatal services alone does not guarantee timely diagnosis and treatment.

In this context, the lower expected number of prenatal visits observed among cases of foreign maternal origin provides complementary information but does not constitute an estimate of access to or adequacy of prenatal care in the source population. For descriptive context, the median number of visits recorded among our cases was below the minimum of eight contacts recommended by the World Health Organization in its model of routine prenatal care (13).

However, visit count alone does not establish adequacy of prenatal care, screening, diagnosis, or treatment, which represent distinct dimensions of congenital syphilis prevention (1, 14–16), nor can it demonstrate mediation of the primary association.

The temporal evolution of rates also requires cautious interpretation. The nonlinear pattern observed indicates that variations during the study period are not adequately described by a constant temporal trend. Hospital-based studies have documented changes in the frequency of maternal syphilis and timing of diagnosis during the COVID-19 pandemic, including a higher proportion of diagnoses at the time of delivery (17). Our analysis was not designed to estimate a pandemic effect or origin-by-time interaction; therefore, variations during this period should not be causally attributed to the pandemic or interpreted as evidence of different temporal trajectories by maternal origin.

Neonatal characteristics comprised a descriptive component of the study. Among recorded cases, prematurity and small-for-gestational-age newborns were observed. Population-based studies have shown associations between maternal syphilis and adverse outcomes such as prematurity, low birth weight, and small-for-gestational-age status, with associations of greater magnitude when maternal infection was untreated (18). Additionally, studies restricted to newborns with congenital syphilis have described prematurity and low birth weight among their findings (19). Because our study did not include a comparison group without congenital syphilis, these characteristics should be interpreted as descriptive findings only and cannot be used to estimate the association between congenital syphilis and the risk of adverse perinatal outcomes.

Study strengths include the prolonged observation period, availability of institutional census denominators of live births according to maternal origin, availability of maternal origin in all recorded cases, and use of a model with flexible temporal structure. Model diagnostics and sensitivity analyses allowed us to assess the stability of the primary association after excluding selected periods and across the different prior specifications evaluated.

The study has several limitations. As a retrospective study conducted in a tertiary-level public referral hospital, the results do not represent population estimates for Quito or Ecuador. Although denominators included all institutional live births, cases identified in the SIP corresponded to hospitalized newborns, recorded regardless of maternal origin. If the probability that a true congenital syphilis case was hospitalized, diagnosed, or recorded in the SIP differed according to maternal origin, differential case ascertainment could bias the estimated rate ratio. The available data did not permit estimation of group-specific probabilities of hospitalization, diagnostic evaluation, or SIP recording; therefore, neither the magnitude nor the direction of this potential bias could be determined. Accordingly, the estimated RR should be interpreted as a contrast in rates of hospital-recorded congenital syphilis cases rather than as a population-level relative risk of congenital syphilis.

Case status was based on the final discharge diagnosis of congenital syphilis recorded in the SIP after clinical assessment during hospitalization, rather than on neonatal seroreactivity alone. Nevertheless, the diagnostic criteria applied to individual cases could not be independently verified, and retrospective reclassification using maternal or neonatal serologic results, clinical findings, and maternal treatment information was not possible. Diagnostic misclassification and temporal variation in clinical or recording practices therefore cannot be excluded. Sensitivity analyses excluding 2021 and the incomplete 2024 period yielded estimates of similar direction and magnitude, although these analyses cannot establish diagnostic validity. The category “foreign origin” grouped heterogeneous countries and contexts and was not equivalent to nationality, migration status, duration of residence, or social vulnerability. Although Venezuelan-origin births constituted the largest component of the foreign-origin denominator, the anonymized case-level dataset did not retain specific country of origin. Therefore, the observed association applies only to the aggregated foreign-origin category and cannot be interpreted as an estimate specific to Venezuelan mothers or mothers from any other country.

Additionally, denominators for Ecuadorian origin were calculated as the difference between the institutional total of live births and those recorded as foreign maternal origin, because historical data sources did not permit separate identification of records with missing or undetermined maternal origin; therefore, some misclassification in these denominators cannot be excluded. The absence of equivalent individual-level covariates for all live births precluded adjustment of the primary association for potential maternal confounders. Data for 2024 covered January–June only; excluding these strata yielded a similar estimate.

Finally, the prenatal care analysis was conducted exclusively among recorded congenital syphilis cases and included a reduced number of foreign maternal origin cases. Therefore, these results describe differences among cases and cannot be used to infer differences in prenatal care among all births to Ecuadorian and foreign-origin mothers or to establish that prenatal care mediates the primary association.

Future studies should use population-based or linked data sources that permit country-specific identification of maternal origin and measurement of migration status, duration of residence, social determinants, and healthcare access. Incorporating maternal screening, timing of diagnosis, and treatment information would also help distinguish differences in underlying maternal infection from potential differences in case ascertainment and identify where gaps occur along the congenital syphilis prevention pathway.

## Data Availability

The dataset is deposited at Mendeley Data (https://doi.org/10.17632/sd8x339hwy.1). Because it contains sensitive health information, access, use, and redistribution are subject to institutional authorization from Hospital Gineco-Obstétrico Isidro Ayora and applicable ethical and confidentiality requirements. The R analysis code, tables, and figures are openly available at Zenodo (https://doi.org/10.5281/zenodo.22239781).
