# Supplementary material for: Hospital-recorded congenital syphilis rates by maternal origin in a referral hospital in Quito, Ecuador, 2009–June 2024: a retrospective observational study
Source: Front Reprod Health. 2026 Oct 30;8:1985888. doi: 10.3389/frph.2026.1985888 (PMC13759845; doi:10.3389/frph.2026.1985888)
Supplement: Supplementary file 1 [file Datasheet1.docx]

**Supplementary Figure S1.** Flow diagram of recorded congenital syphilis case selection for the primary analysis. Institutional live-birth denominators were obtained from the institutional census, whereas congenital syphilis cases were identified among hospitalized newborns recorded in the Perinatal Information System (SIP). All 255 hospital-recorded cases had available maternal origin and corresponding institutional live-birth denominators; no eligible recorded cases were excluded from the primary analysis.

**Supplementary Figure S2.** *Posterior predictive checks of the primary Bayesian Poisson model.*

*Note: In each panel, the orange line represents the observed value, and the distribution represents values obtained from data replicated by the model using posterior_predict().*

### Supplementary Table S1. Annual distribution of institutional live births by maternal origin, 2009–June 2024

| Year | Live births to mothers of Ecuadorian origin, n | Live births to mothers of foreign origin, n | Total institutional live births, n |
| --- | --- | --- | --- |
| 2009 | 11,265 | 207 | 11,472 |
| 2010 | 11,392 | 226 | 11,618 |
| 2011 | 10,804 | 245 | 11,049 |
| 2012 | 9,507 | 235 | 9,742 |
| 2013 | 9,731 | 249 | 9,980 |
| 2014 | 8,710 | 213 | 8,923 |
| 2015 | 7,378 | 321 | 7,699 |
| 2016 | 5,757 | 251 | 6,008 |
| 2017 | 5,312 | 371 | 5,683 |
| 2018 | 4,717 | 414 | 5,131 |
| 2019 | 3,681 | 531 | 4,212 |
| 2020 | 4,463 | 773 | 5,236 |
| 2021 | 3,657 | 561 | 4,218 |
| 2022 | 3,851 | 454 | 4,305 |
| 2023 | 3,671 | 437 | 4,108 |
| 2024 | 2,146 | 212 | 2,358 |

***Note:*** *Denominators correspond to the institutional census of live births presented by year and maternal origin category. For each period, the sum of live births to mothers of Ecuadorian and foreign origin equals the total institutional live births. Data for 2024 include only January through June.*

### Supplementary Table S2. Annual distribution of institutional live births classified as foreign maternal origin, by recorded country of origin, 2009–June 2024

| **Year** | **Venezuela** | **Colombia** | **Cuba** | **Peru** | **Other countries** | **Total** |
| --- | --- | --- | --- | --- | --- | --- |
| **2009** | 6 | 135 | 41 | 17 | 8 | 207 |
| **2010** | 8 | 144 | 43 | 28 | 3 | 226 |
| **2011** | 9 | 138 | 61 | 31 | 6 | 245 |
| **2012** | 12 | 141 | 58 | 21 | 3 | 235 |
| **2013** | 15 | 151 | 55 | 25 | 3 | 249 |
| **2014** | 18 | 125 | 48 | 18 | 4 | 213 |
| **2015** | 21 | 168 | 59 | 23 | 50 | 321 |
| **2016** | 58 | 105 | 29 | 15 | 44 | 251 |
| **2017** | 196 | 97 | 22 | 9 | 47 | 371 |
| **2018** | 293 | 59 | 18 | 5 | 39 | 414 |
| **2019** | 422 | 55 | 5 | 7 | 42 | 531 |
| **2020** | 670 | 63 | 12 | 4 | 24 | 773 |
| **2021** | 463 | 64 | 9 | 7 | 18 | 561 |
| **2022** | 355 | 58 | 7 | 9 | 25 | 454 |
| **2023** | 339 | 59 | 7 | 8 | 24 | 437 |
| **2024** | 151 | 45 | 1 | 3 | 12 | 212 |
| **Total** | **3,036** | **1,607** | **475** | **230** | **352** | **5,700** |

***Note:*** *Country-specific information was available only for aggregated institutional live-birth denominators. The anonymized individual-level analytic dataset retained maternal origin only as Ecuadorian or foreign; therefore, country-specific congenital syphilis case counts and rates could not be estimated. ‘Other countries’ combines countries with small numbers of institutional live births. Data for 2024 include January through June only.*

### Supplementary Table S3. Comparison of fit and dispersion of exploratory frequentist models

| Model Comparison | Log-likelihood | AIC | BIC | χ² LRT | df | P | Pearson Dispersion |
| --- | --- | --- | --- | --- | --- | --- | --- |
| Maternal origin only vs. maternal origin + linear year | −80.0260 | 166.0519 | 170.4491 | 9.5035 | 1 | 0.0021 | 1.9318 |
| Maternal origin + linear year vs. maternal origin × year interaction | −79.7165 | 167.4331 | 173.2960 | 0.6188 | 1 | 0.4315 | 2.0079 |
| Maternal origin + linear year vs. natural spline of year | −73.5269 | 157.0538 | 164.3825 | 12.9981 | 2 | 0.0015 | 1.5425 |

***Note:*** *Log-likelihood, AIC, BIC, and Pearson dispersion values correspond to the second model indicated in each comparison. The LRT statistic compares the two models specified in each row. AIC, Akaike information criterion; BIC, Bayesian information criterion; LRT, likelihood ratio test; df, degrees of freedom.*

### Supplementary Table S4. MCMC diagnostics of the primary Poisson model

| Diagnostic | Chain | Value |
| --- | --- | --- |
| Maximum Rhat | All | 1.0021 |
| Minimum bulk ESS | All | 1,746 |
| Minimum tail ESS | All | 2,501 |
| Divergences | All | 0 |
| Max treedepth exceedances | All | 0 |
| Minimum E-BFMI | All | 0.8055 |
| E-BFMI | Chain 1 | 0.8532 |
| E-BFMI | Chain 2 | 0.8631 |
| E-BFMI | Chain 3 | 0.8682 |
| E-BFMI | Chain 4 | 0.8055 |

***Note:*** *ESS, effective sample size; E-BFMI, Bayesian fraction of missing information.*

### Supplementary Table S5. Posterior predictive checks of the primary Poisson model

| Metric | Value |
| --- | --- |
| Observed variance | 59.32 |
| Median replicated variance | 51.47 |
| 95% posterior predictive interval—lower | 32.51 |
| 95% posterior predictive interval—upper | 80.26 |
| P(replicated variance ≥ observed) | 0.2687 |
| Observed maximum | 26 |
| P(replicated maximum ≥ observed) | 0.4298 |
| 2021–Ecuadorian origin: observed | 24 |
| 2021–Ecuadorian origin: posterior median | 11 |
| 2021–Ecuadorian origin: 95% PPI lower | 5 |
| 2021–Ecuadorian origin: 95% PPI upper | 18 |
| 2021–Ecuadorian origin: P(y_rep ≥ observed) | 0.0014 |
| 2021–Ecuadorian origin: observed percentile | 0.9990 |

***Note:*** *PPC, posterior predictive check; PPI, posterior predictive interval; y_rep, posterior replicated count.*

### Supplementary Table S6. Leave-one-out cross-validation diagnostics.

#### Panel A. Summary

| Metric | Value |
| --- | --- |
| ELPD-LOO | −82.88 |
| Standard error of ELPD | 9.66 |
| p-LOO | 8.4 |
| Maximum Pareto-k | 0.695 |
| Stratum with maximum Pareto-k | 2021–Ecuadorian origin |

#### Panel B. Distribution of Pareto-k values

| Pareto-k Classification | Number of Strata |
| --- | --- |
| k ≤ 0.5 | 30 |
| 0.5 < k < 0.7 | 2 |
| 0.7 ≤ k ≤ 1 | 0 |
| k > 1 | 0 |

#### Panel C. Ten strata with the highest Pareto-k values

| Year | Maternal Origin | Cases | Live Births | Pareto-k |
| --- | --- | --- | --- | --- |
| 2021 | Ecuadorian | 24 | 3,657 | 0.695 |
| 2012 | Ecuadorian | 24 | 9,507 | 0.561 |
| 2010 | Ecuadorian | 26 | 11,392 | 0.465 |
| 2023 | Ecuadorian | 6 | 3,671 | 0.370 |
| 2009 | Ecuadorian | 22 | 11,265 | 0.353 |
| 2014 | Ecuadorian | 16 | 8,710 | 0.353 |
| 2024 | Ecuadorian | 3 | 2,146 | 0.341 |
| 2013 | Ecuadorian | 10 | 9,731 | 0.335 |
| 2023 | Foreign | 5 | 437 | 0.308 |
| 2018 | Foreign | 5 | 414 | 0.301 |

***Note:*** *ELPD-LOO, expected log predictive density via leave-one-out cross-validation; SE, standard error; p-LOO, effective number of parameters; Pareto-k, PSIS-LOO diagnostic. Of 32 strata, 30 had k ≤ 0.5 and two had values between 0.5 and 0.7; no values ≥0.7 were observed. The maximum Pareto-k was 0.695 and corresponded to the 2021–Ecuadorian origin stratum.*

### Supplementary Table S7. Temporal sensitivity of the primary Poisson model.

| Panel | Scenario | Strata | RR | 95% CrI | P[RR > 1] |
| --- | --- | --- | --- | --- | --- |
| Reference | Primary model | 32 | 2.47 | 1.69–3.51 | 1.0000 |
| A | Excluding both 2021 strata | 30 | 2.83 | 1.86–4.17 | 1.0000 |
| B | Excluding both 2024 strata | 30 | 2.45 | 1.65–3.47 | 1.0000 |

***Note:*** *Panel A excludes both 2021 strata; Panel B excludes both 2024 strata. The reference model retains all 32 strata. CrI, credible interval.*

### Supplementary Table S8. Sensitivity of the primary model to prior distributions for the maternal origin coefficient.

| Scenario | Prior for Maternal Origin | RR | 95% CrI | P[RR > 1] | Maximum Rhat | Divergences | ELPD-LOO |
| --- | --- | --- | --- | --- | --- | --- | --- |
| Primary | Normal(0, 1) | 2.47 | 1.69–3.51 | 1.0000 | 1.0021 | 0 | −82.88 |
| More regularized | Normal(0, 0.5) | 2.27 | 1.57–3.19 | 1.0000 | 1.0018 | 0 | −82.70 |
| Less regularized | Normal(0, 1.5) | 2.52 | 1.75–3.52 | 1.0000 | 1.0009 | 0 | −82.87 |

***Note:*** *Each scenario modifies only the standard deviation of the Normal prior centered at zero for the maternal origin coefficient. CrI, credible interval; ELPD-LOO, expected log predictive density via leave-one-out cross-validation.*
